# Aptasensors Based on Whispering Gallery Mode Resonators

**DOI:** 10.3390/bios6030028

**Published:** 2016-07-16

**Authors:** Gualtiero Nunzi Conti, Simome Berneschi, Silvia Soria

**Affiliations:** Istituto di Fisica Applicata Nello Carrara (IFAC CNR), Via Madonna del Piano 10, 50019 Sesto Fiorentino, Firenze, Italy; g.nunziconti@ifac.cnr.it (G.N.C.); s.berneschi@ifac.cnr.it (S.B.)

**Keywords:** aptamers, whispering gallery mode resonators, microspheres, integrated rings, optical sensors

## Abstract

In this paper, we review the literature on optical evanescent field sensing in resonant cavities where aptamers are used as biochemical receptors. The combined advantages of highly sensitive whispering gallery mode resonator (WGMR)-based transducers, and of the unique properties of aptamers make this approach extremely interesting in the medical field, where there is a particularly high need for devices able to provide real time diagnosis for cancer, infectious diseases, or strokes. However, despite the superior performances of aptamers compared to antibodies and WGMR to other evanescent sensors, there is not much literature combining both types of receptors and transducers. Up to now, the WGMR that have been used are silica microspheres and silicon oxynitride (SiON) ring resonators.

## 1. Introduction

Ellington and Szostak [1] developed for the first time an in vitro selection technique that allowed the discovery of oligonucleotides or peptides that bind to specific targets with high specificity and affinity. Since then, the interest in aptamers has increased significantly [2,3,4] because of their advantages compared to other biological receptors. Among these properties, we can enumerate the size, the synthesis and the stability. Additionally, the aptamer-analyte bound might be disrupted by very small changes.

Whispering gallery mode based resonators (WGMR) have attracted much attention in the biomedical sensing field [5,6], including aptamer based sensors for thrombin detection [7,8], vascular endothelial growth factor [8] and toxins such as aflatoxin M1 [9]. WGMR have different morphologies with particular spectral features such as including narrow linewidth, high stability, and tunability. Regarding sensing applications, the most important properties are the ultrahigh quality factor *Q* and long lifetime of WGMR, where the change in *Q* or the shift resonant wavelength is used for measuring the change of parameters in the surrounding environment or binding event on the WGMR surface. WGMR are a valid alternative to other evanescent wave (EW) sensors, such as surface plasmon resonators (SPR) [10]. The feasibility of WGMR for detecting of single virions has been demonstrated recently [11]. Silica WGMR have two important advantages when compared to other EW sensors: a significantly longer penetration depth and a well established surface chemistry for silica. The combination of WGMR and covalent chemistry will be able to provide very accurate biochemical sensors.

Thrombin is a powerful vasoconstrictor that is involved in many diseases, like atherosclerosis and thrombosis. Vascular endothelial growth factor (VEGF) is a signaling protein that regulates angiogenesis and its normal function is the formation of new blood vessels. VEGF, when overexpressed, can also cause vascular diseases and enhance metastasis if the tumor cells can express it. Detecting these blood proteins in laboratory and clinical measurements is time consuming and costly, because there are not always available antibodies. Regarding toxins, aflatoxin M1 (AFM1) is a milk contaminant and potent carcinogen (European Commission regulation, EC No. 1881/2006) limiting the maximum allowable concentration of Aflatoxin in milk products to 50 ng/kg. Thus, aflatoxin contamination represents a serious threat to human health and, in economical terms, a loss for the dairy industry. Presently, the screening procedure involves Enzyme-LinkedImmunoSorbent Assay (ELISA) tests [12], and the suspicious samples need further investigations with High-Performance Liquid Chromatography (HPLC) tests [13], which are costly and time-consuming processes.

The focus of this review is on biochemical sensing using WGMR as transducers and aptamers as biochemical receptors. The aim of our review is also to show the feasibility of WGMR aptasensors in medical diagnosis and food safety controls. However, direct bacteria detection is quite challenging when using EW sensors due to their large dimensions [14]. 

Usually, the biological recognition elements (BRE) are antibodies, streptavidin, enzymes and aptamers; these BRE bind specifically to their corresponding analytes: antigens, biotin/biotinylated proteins, amino acids, and proteins. Antibodies and antigens are highly complementary, and, as a consequence, highly specific. Streptavidin shows a high specificity only to biotin, but for sensing other analytes there is the need to biotinylate the analytes first, adding an extra chemical step. Enzymes are able to catalyze a large number of reactions, they are also able to detect substrates, inhibitors and products of the catalysis. The main advantages are specificity and catalytic activity. However, enzyme activity largely depends on the environment. Aptamers are engineered molecules, with high specificity and affinity towards the target analyte (proteins, cells, small molecules, etc.). The binding may be disrupted by very small changes. However, despite the superior performances of aptamers compared to antibodies and WGMR to other EW sensors, there is not much literature combining both types of receptors and transducers. Up to now, the WGMR that have been used are silica microspheres and silicon oxynitride (SiON) ring resonators.

## 2. Materials and Methods

### 2.1. Surface Functionalisation

The surface functionalization is the most important step in the production of accurate biosensors. The BRE layer has to be thinner than the evanescent tail and smooth for preserving the high *Q* of the WGMR . As mentioned in the introduction, there is a wide choice of BREs. Among the various techniques, covalent silane chemistry is the one used in WGMR aptasensors.

The first silica microspherical WGMR aptasensor paper was published by Fan et al. [7]. Silica microspheres were functionalized in dry conditions using 3-Aminopropyltrimethoxysilane (3-APS, 97%, Sigma Aldrich, St. Louis, MO, USA) whereas in the second one, published by Pasquardini et al. [8], the authors used 3-mercaptopropyltrimethoxysilane (MPTMS, 99%, Gelest) in wet conditions. SiON ring resonators were also functionalized in wet conditions using a 3-glycidoxypropyl methyldiethoxy silane (GPTMS) solution [9].

#### 2.1.1. Materials and Silica Microspherical WGMR Functionalisation

##### Dry Protocol

3-Aminopropyltrimethoxysilane (3-APS, 97%), 1–4 phenylene diisothiocyanate (PDC) Pyridine, *N,N*-dimethyl formamide (DMF), anhydrous ethanol, acetone and thrombin from human plasma (38 kD MW) from Sigma Aldrich are used without any further purification. The assay of all chemicals is 98% except pyridine, which is 99%.

The thrombin aptamer (5′-CCAACCCAACGGTTGGTGTGGTTGG-3′) [7] and the non-sense apatamer sequence (5′-TATGAATTCAATCCGTCGAGCAGAGTT-3′) were also used without further purification. Both aptamer sequences were modified at the 5′ end with amino groups and were purchased from Invitrogen.

After oxygen plasma cleaning (plasma cleaner PDC-32G, Harrick, Ossining, NY, USA) for 10 min, the WGMR were exposed to vapor silanization for 1 h inside a desiccator. 3-APS was used pure. Silanized WGMR were then annealed at 160 °C for 20 min. Afterwards, WGMR followed a two hours incubation in a 1% (*w*/*w*) PDC solution in a 10/90 (*v*/*v*) pyridine DMF solution. This step was needed for cross-linking the surface amine groups to the protein amine groups [15]. The last two steps were rinsing five times with anhydrous ethanol and acetone, respectively, one hour incubation were in 2 mM anti-thrombin aptamer in carbonate buffer (pH = 8.3). The storage procedure was done at room temperature.

##### Wet Protocol

The following chemicals were used in the wet protocol: 3-mercaptopropyltrimethoxysilane (MPTMS, 99%, Gelest), toluene anhydrous (99.8%, Sigma Aldrich) and toluene (Sigma-Aldrich); purified Human Thrombin (0.3 mg/mL, Bioultra, THR, Sigma Aldrich), purified VEGF165 (0.3 mg/mL, Sigma Aldrich); Bovine Serum Albumin (BSA, Thermo Scientific) and Prionex^®^ solution (Branch Pentapharm, Switzerland). Two different buffer solutions were used: the Coupling Buffer (Na_2_CO_3_/NaHCO_3_ 0.5 M, pH = 9, CB) for aptamer incubation and the BioRecognition Buffer (Tris 50 mM, EDTA 1 mM, MgCl_2_ 1 mM, KCl 150 mM pH = 7.4, BRB) was used in the thrombin incubation whereas for VEGF detection the biorecogniton buffer was a Tris-HCl 20 mM buffer solution with a pH = 7.4. β-mercapto-ethanol (MP-ET, Sigma-Aldrich) was used for passivating the remaining active sites in the modified WGMR surface. For activated WGMR regeneration, a 50 mM NaOH solution was used. The aptamer sequences are HPLC purified (IDT Integrated DNA Technologies, Leuven, Belgium).

The sequences of the Thrombin Binding Aptamer (TBA) are:

- 5′-HO-(CH_2_)_3_-S-S-(CH_2_)_3_- GGT TGG TGT GGT TGG-3′ (TBA-15)
- 5′-HO-(CH_2_)_3_-S-S-(CH_2_)_3_-AG TCC GTG GTA GGG CAG GTT GGG GTG ACT-3′ (TBA-29)

The sequence of the non specific aptamer is:

- 5′-HO-(CH_2_)_3_-S-S-(CH_2_)_3_-CCG TCG AGC AGA GTT-3′ (NS, Non Sense)

The sequence specific for VEGF165 detection is:

- 5′-HO-(CH_2_)_3_-S-S-(CH_2_)_3_-CC GTC TTC CAG ACA AGA GTG CAG GG-3′ (hereafter called VEGF-25)

MPTMS has thiol groups instead of amine groups, and it was chosen for binding the dithiol groups at 5′ end of the aptamer sequences. The first step is the cleaning and oxidizing one, using piranha solution (H_2_SO_4_:H_2_O_2_ at 4:1 *v*/*v* concentration) for 3 min, followed by a washing one using MilliQ water plus a drying step in an oven at 110 °C for two hours or more. The activated WGMR were then silanized at 60 °C using a MPTMS toluene anhydrous solution (0.0025% *v*/*v*) for a total time of about 10 min. Silanized WGMR were placed in a chemical hood for toluene rinsing and then drying.

The aptamer sequences were immobilized on silanized WGMR adapting Hilliard’s procedure described in [16]. In summary, this procedure consists in a short thermal treatment at 95 °C followed by cooling step in ice. The silanized WGMR were then incubated in an orbital shaker for 2 h in a 10 μM aptamer solution in CB buffer, followed by a washing step in CB buffer. Finally, the passivation step at room temperature was performed in 1 mM MP-ET in CB buffer for 2 h, this step reduces the aptamer density by a factor of 22. This last step enhances the target recognition, since it allows the aptamer structuration and it also blocks the residual free binding sites.

#### 2.1.2. Materials and SiON WGMR Functionalization

A wet silanization protocol was used for functionalizing these sensors. First the transducer were cleaned with a Piranha solution, afterwards the samples were silanized by immersion in 0.01% *v*/*v* of GPTMS (3-glycidoxypropyl methyldiethoxy silane) solution in anhydrous toluene at 60 °C for 10 min. Then an amino-terminated DNA-aptamer (5′-NH_2_-(CH_2_)_6_-GT TGG GCA CGT GTT GTC TCT CTG TGT CTC GTG CCC TTC GCT AGG CCC ACA-3′) at 100 μM in phosphate buffer (50 mM, ionic strength 300 mM, pH 8) was incubated on silanized surfaces for 2 h. The aptameric anti-aflatoxin DNA sequence with a kD of 10 nM was identified by NeoVentures Biotechnology Inc. [17]. The aminomodified sequence is HPLC purified and was purchased from IDT Integrated DNA Technologies (Leuven, Belgium). Finally, an ethanolamine passivation at 1 mM for 30 min was applied.

### 2.2. Experimental Set-up

#### 2.2.1. Experimental Set-ups for Microspheres Based Aptasensors

The easiest microspherical WGMR fabrication technique is based on a fusion method. There are two techniques widely use: CO_2_ laser irradiation [18,19] or melting by electrical arcs discharged by a fiber fusion splicer [20,21] of a silica fiber tip. The partial melting of the silica and surface tension produce a spherical shape. The microspheres used in sensing have a diameter that can oscillate from 50 of 260 μm [7,22,23] and are stored under vacuum at room temperature, in order to avoid contamination.

**Figure 1:**
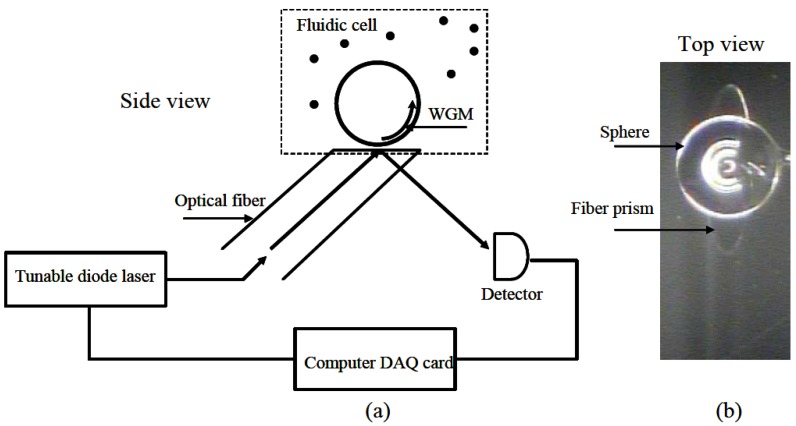
(**a**) Schematic of the microsphere and fluidic cell (side view); (**b**) top view of the fiber prism with a microsphere. Reprinted with permission from [7] © 2006 MDPI.

Sample delivery is done using fluidic cells. A small fluidic cells were fabricated by using an angle-polished fiber prism [7] as both the cell bottom enclosure and coupling system. Figure 1 shows the angle-polished fiber prism approach.

**Figure 2:**
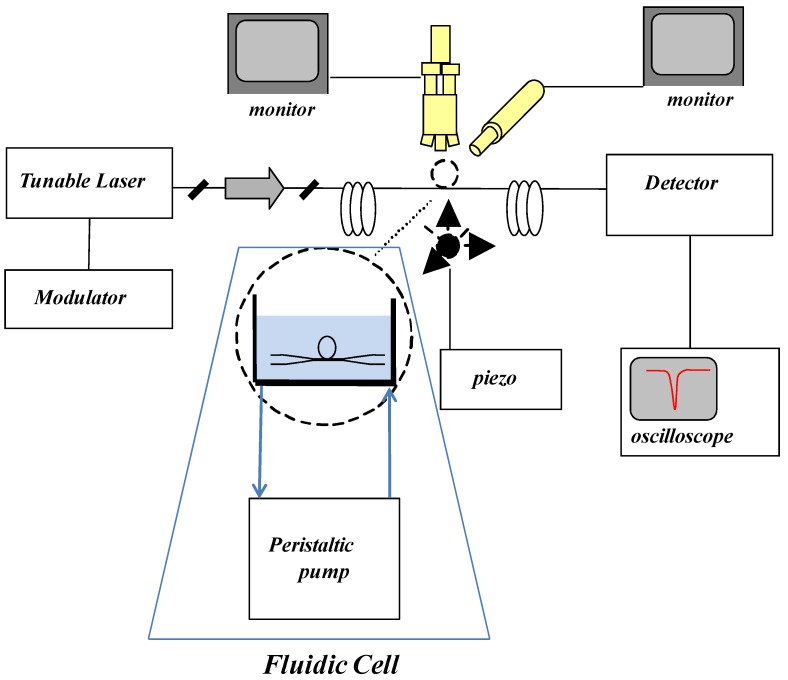
A schematic diagram of the experimental arrangement. Reprinted with permission from [24] © 2012 MDPI.

The laser light can also be coupled to the WGM resonator using a tapered fiber. Figure 2 shows another microfluidic flow system [24]. The fluidic cell is open and it has a volume of about of 350 μL with one inlet connected to a peristaltic pump and an outlet connected to a reservoir. The fluidic loop is open for constant washing of the protein excess. The tapered fiber was placed at the bottom of the cell in parallel to the flow. The experimental flow rate was kept at 167 μL/min [8]. In order to avoid the non selective binding of proteins either in the cell walls or in the taper, the microfluidic flow system is passivated.

A tunable narrow linewidth laser is used to excite the resonant wavelengths. Such a laser can be finely swept slowly with high accuracy. The light transmitted or reflected through the coupler-WGMR system is monitored into an oscilloscope. The resonances are spectral dips that can shift when the size and/or the refractive index of the resonators change.

#### 2.2.2. Experimental Set-ups for Racetrack Microring Based Aptasensors

For the microring fabrication, SiON films were deposited by plasma enhanced chemical vapor deposition (PECVD) on 6-in, 625 μm thick crystalline Si wafers with a 4 μm thick buffer oxide layer. The waveguides, ring resonators and directional couplers structures were fabricated using standard UV-lithography and reactive-ion etching techniques. An annealing step of 1.5 h at 1050 °C was also performed in order to remove hydrogen bonds from the material and decrease the losses of the structures. Finally, the processed wafers were covered with a 1 μm TEOS film, used as a cladding layer.

**Figure 3:**
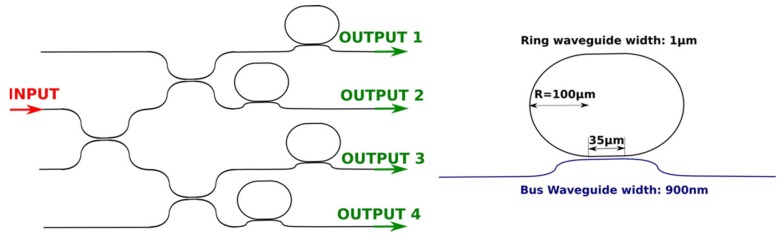
A schematic diagram of the microring based biosensor. **Left**: the input light is split into the four ring resonators using directional couplers with a gap of 600 nm. **Right**: zoomed in image of the ring resonator structure. The gap and coupling length between the bus waveguide and the resonator are 600 nm and 35 μm, respectively. The radius of the resonators is 100 μm. Reprinted with permission from [25] © 2015 Elsevier.

In order to create the sensors, the cladding layer was opened through a BHF wet etching. This allows the functionalization of the rings as well as the sensing measurements through their exposure to the ambient and to the molecules to be analyzed [25]. Figure 3 shows a sketch of the sensor configuration that was coupled to a PDMS microfluidic flow cell, with a chamber of about 0.5 μL of volume, connected to a VICI M6 liquid handling pump. A detailed characterization of the racetrack microring resonators can be found at [9].

## 3. Results

### 3.1. Microspherical Aptasensors for Thrombin and VEGF

After reaching thermal equilibrium, the analyte is injected into the BRB. The wavelength first decreases (blue shift) due to thermal contraction of the microsphere, afterwards resonant wavelength increases till it saturates with time.

**Figure 4:**
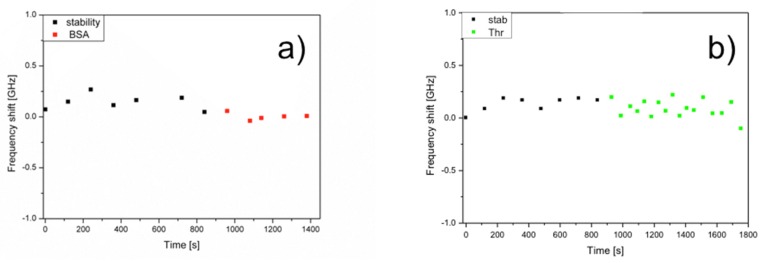
Control experiments: (**a**) whispering gallery mode resonator (WGMR) response of a Thrombin Binding Aptamer (TBA)-15 sensing surface for 0.3 mg/mL Bovine Serum Albumin (BSA) injected into the flow cell and (**b**) sensorgram using a non sense aptamer sensing surface for 0.3 mg/mL thrombin.

The following control experiments were performed, one was performed injecting a BSA solution of similar concentration into the flow cell with the right aptamer bound on the WGMR surface; the second one was performed using a non-sense aptamer bound on the spheres surface but injecting thrombin into the flow cell [7,8]. The BSA spikes had no effect on the WGMR aptasensor response (Figure 4a). Figure 4b shows that thrombin spikes had no effect on the non-sense aptamer bound to the WGMR surface.

**Figure 5:**
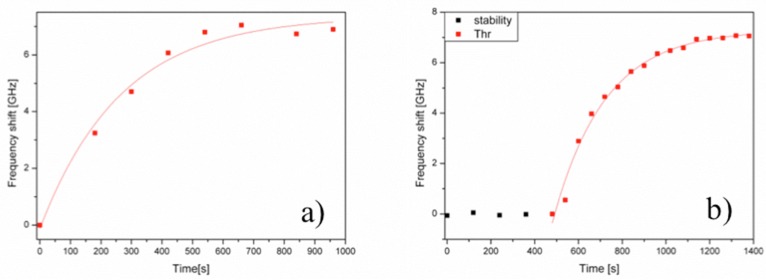
(**a**) WGMR response with TBA-15 modified resonator following the addition of thrombin in buffer (**b**) Sensorgram of thrombin binding to a WGMR immobilized with TBA-15 in 1:10 non-filtered human serum, following the addition of thrombin.

Zhu et al. performed one single specific bioassay for TBA-15 in buffer. Pasquardini et al. performed two specific binding assays for TBA-15 and TBA-29, both in buffer and in a complex matrix (1:10 human serum solution in buffer (*v*/*v*) without any filtering). Figure 5a shows the results for TBA-15 in buffer and in serum. The same authors repeated the same procedure for the VEGF-25 DNA-aptamer. Both authors first performed one control analysis consisting of injecting BSA of similar concentration into the flow cell with the aptamer immobilized on the WGMR surface. Similar results were obtained after spiking 5 μL of 0.3 mg/mL of VEGF165 into the flow cell, reaching equilibrium in 15 min.

**Figure 6:**
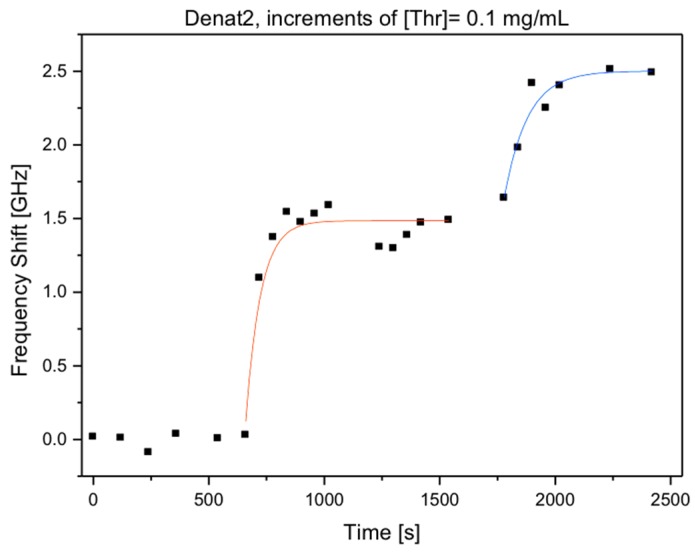
Sensorgram of thrombin binding to a regenerated WGMR immobilized with TBA-15, following the addition of thrombin (two identical additions of 5 μL of 0.1 mg/mL of thrombin).

The same group also tested the capability of reusing the microspheres after treatment with NaOH. The sensor regeneration was done by 50 mM NaOH solution and the WGMR aptasensors was then used for another detection cycle. The binding capacity was maintained as shown in figure 6. Figure 6 shows a sensorgram of a microsphere regenerated twice with two injections of 5 μL of 0.1 mg/mL of thrombin. As expected, due to lower concentration of thrombin, equilibrium was not reached after the first injection.

### 3.2. Racetrack Ring Aptasensors for Aflatoxin M1

The Aflatoxin-sensing measurements were performed in a microfluidic chamber filled with a buffer solution mainly based on 2-(Nmorpholino) ethanesulfonic acid (MES) and dimethyl sulfoxide (DSMO). The DSMO is needed to dissolve the aflatoxin. Then aflatoxin AFM1 solutions were injected into the chamber, and the resonance shift was monitored in real time. Finally, we injected again the buffer solution. All measurements were done with a flow of 3 μL/min.

Three solutions with different concentrations were flown into the chamber, one at 12.5 nM, one at 25 nM and the last at 50 nM. After each Aflatoxin injection, two or three Glycine (100 mM glycine-HCl, pH 2) injections were effectuated, in order to detach the toxin from the sensor and regenerate the aptamers. The regeneration performances of Glycine concerning this aptamer were already demonstrated in [26,27].

**Figure 7:**
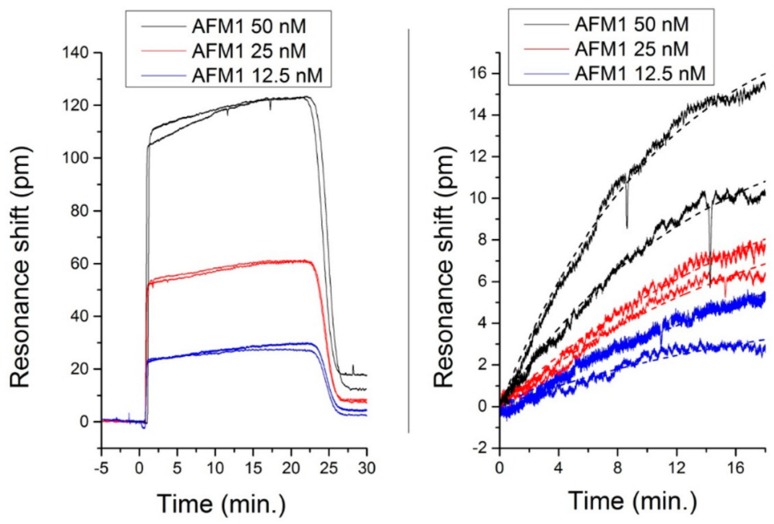
(**left**) Sensorgram for three different concentrations of alflatoxin M1 (AFM1). The high step-like response is due to the refractive index mismatch produced by the small content of Dimethyl sulfoxide (DMSO) in the solution; (**right**) Specific binding sensorgrams obtained from the curves in (**left**) by subtracting the bulk shift induced by the DMSO content. The dashed curves are exponential fittings for the evaluation of the rate constants and of the initial slopes [27]. Reprinted with permission from [9] © 2015 MDPI.

Figure 7 shows the sensorgrams for the three concentrations of AFM1, demonstrating the reproducibility and reusability of the sensor for AFM1. The binding effect was observed down to a minimum concentration of 1.58 nM [25].

## 4. Discussion

The resonant wavelength shifts are calculated as the variation of the radius of the microsphere R and its index of refraction n_s_, using the following expression: *Δλ/λ = ΔR/R* + *Δn_s_/n_s_*. However, the expression above is not valid for complex molecules binding to a surface [18]. In this case, the resonance shift is due to their excess of polarizability, α***_ex_***, and it can be described using the following equation:

δλ/λ = α_ex_σ/[ε_0_(n_s_^2^ − n^2^_m_)R] (1)

where *σ* is the surface density of molecules forming a layer, and *n_s_* and *n_m_* are the refractive index of the sphere and the medium, respectively; and ε_0_ is the vacuum permittivity.

Thrombin excess of polarizability is 4πε_0_ × 2.1 × 10^−21^ cm^3^, R = 130 μm (average microsphere radius of Pasquardini’s experiments), n_s_ = 1.49 (silica index of refraction), n_m_ = 1.33 (buffer index of refraction) and δλ = 14.6 pm [8], the thrombin surface density is calculated to be 3.14 × 10^12^ cm^−2^, which is close to a compact layer. Thrombin is a roughly spherical molecule of about 4.6 nm of diameter [28], so its projected area is about 17 nm^2^ [7] and its binding kinetics can be successfully modeled by treating thrombin as a sphere. The maximum fractional area coverage is estimated to be 0.55 for random packing of spheres [29] which means that σ_max_ = 3.24 × 10^12^ cm^−2^ for thrombin. Thus, the thrombin layer is about the theoretical level. The VEGF165 calculated surface density is about 1.98 × 10^12^ cm^−2^. In Zhu et al., the thrombin surface density was about 5.4 × 10^12^ cm^−2^ for a maximum δλ of about 34 pm.

Since desorption was not observed in our experiments, binding of kinetics could be described using a simple differential equation that neglects the dissociation rate constant. The response of the the WGMR aptasensor is then a simple exponential [30]:

Δν = ν_opt_ δR_max_/R[1 − exp(−*k_a_*Ct)], δR_max_ = (Δν_max_ R)/ν_opt_ (2)

where ν_opt_ is the optical frequency, C is the concentration and *k_a_* is the association constant. It should be noted that transport phenomena and steric hindrance were also neglected [31]. From the experimental data, Δν_max_ is 7.44 ± 0.22 GHz and assuming that binding is saturated with the used concentration, we obtained δR_max_ = 2.49 ± 0.07 nm. This is consistent with monolayer coverage confirming the AFM measurements (not shown in here). The association constant *k_a_* is about 8.8 ± 0.4 × 10^4^ M^−1^·s^−1^ for TBA-15 and about 2.2 × 10^4^ M^−1^·s^−1^ for TBA-29, which are in good accordance with published results for TBA-15 [32] and TBA-29 [33].

## 5. Conclusions

Several groups have demonstrated the potential of the WGMR based aptasensors in medicine and also in food safety. Microspherical WGMR have been used in the first demonstration of aptasensing. Microspheres have been used for detection of blood proteins such as thrombin (TBA-15 and TBA-29) and VEGF, whereas racetrack microrings have been used for detection of pathogens such as aflatoxin M1. WGMR aptasensors are real time sensors with high specificity and good reversibility that can be used for determining binding kinetics.

## Abbreviations

The following abbreviations are used in this manuscript:

WGMR: Whispering gallery mode resonators
SiON: silicon oxynitrid
SPR: surface plasmon resonators
VEGF: Vascular endothelial growth factor
AFM1: aflatoxin M1
ELISA: Enzyme-LinkedImmunoSorbent Assay
HPLC: High-Performance Liquid Chromatography
APS: Aminopropyltrimethoxysilane
MPTMS: mercaptopropyltrimethoxysilane
GPTMS: glycidoxypropyl methyldiethoxy silane
PDC: phenylene diisothiocyanate
DMF: dimethyl formamide
CB: Coupling Buffer
MB: Modification Buffer
BRB: BioRecognition Buffer
TBA: Thrombin Binding Aptamer
PECVD: plasma enhanced chemical vapor deposition
MES: 2-(Nmorpholino) ethanesulfonic acid
DSMO: dimethyl sulfoxide
